# CHIC: A machine learning framework for inferring the presence of high‐risk clonal hematopoiesis using complete blood count data from 431,531 UK Biobank participants

**DOI:** 10.1002/hem3.70169

**Published:** 2025-07-03

**Authors:** William G. Dunn, Isabella Withnell, Muxin Gu, Pedro Quiros, Sruthi Cheloor Kovilakam, Ludovica Marando, Sean Wen, Margarete A. Fabre, Irina Mohorianu, Dragana Vuckovic, George S. Vassiliou

**Affiliations:** ^1^ Cambridge Stem Cell Institute University of Cambridge Cambridge UK; ^2^ Department of Haematology University of Cambridge Cambridge UK; ^3^ Department of Haematology Cambridge University Hospitals NHS Trust Cambridge UK; ^4^ Division of Biosciences University College London London UK; ^5^ Department of Biochemistry and Molecular Biology, Instituto Universitario de Oncología (IUOPA) Universidad de Oviedo Oviedo Spain; ^6^ Department of Hematology Mayo Clinic Rochester Michigan USA; ^7^ Centre for Genomics Research, Discovery Sciences Biopharmaceuticals R&D, AstraZeneca Cambridge UK; ^8^ Department of Epidemiology and Biostatistics School of Public Health, Imperial College London, Faculty of Medicine London UK

Clonal hematopoiesis (CH) is an age‐related phenomenon that arises when a hematopoietic stem cell acquires a somatic driver mutation (i.e., one that increases its fitness), leading to clonal expansion of the cell and its progeny.^1^, ^2^ Large population‐based studies have revealed that the most commonly mutated genes in CH are involved in epigenetic regulation (*DNMT3A*, *TET2*, and *ASXL1*), signal transduction (*JAK2*, *GNB1*), DNA damage response and apoptosis (*TP53*, *PPM1D*), and splicing (*SF3B1*, *SRSF2*, and *U2AF1*).^1^, ^2^, ^3^, ^4^, ^5^, ^6^ The prevalence of CH increases with advancing age to affect at least 20% of those over 70 years, in whom the phenomenon is almost universally detectable when deep sequencing approaches are employed.^1^, ^2^, ^3^, ^4^, ^5^, ^6^

A hallmark of CH is the associated increased risk of incident myeloid neoplasms (MNs), a molecularly heterogeneous group of blood cancers that include acute myeloid leukemia, myelodysplastic syndromes (MDSs), and myeloproliferative neoplasms (MPNs). Recent advances have led to the development of predictive tools that estimate the risk of progression from CH to MN,^7^, ^8^ such that individuals at high risk can be identified and prioritized for clinical follow‐up. As CH precedes the development of MN by several years,^1^, ^2^, ^3^, ^7^, ^9^, ^10^ this provides a window during which high‐risk clones could be intercepted and targeted to avert or delay the development of MN.

A key impediment to prospective myeloid cancer prevention programs is the lack of a scalable test to identify individuals with CH. At present, CH is identified by next‐generation sequencing (NGS) of blood DNA targeted to a panel of genes recurrently mutated in MN. However, NGS is not performed in routine clinical practice and is impractical and costly to perform at scale. An alternative approach is to leverage low‐cost, scalable, routine clinical tests to identify the individuals most likely to harbor CH, who can then be prioritized for sequencing. The complete blood count (CBC) is an inexpensive, routine clinical test, and CBC indices such as the red cell distribution width (RDW) and mean cell volume are known to be associated with progression from CH to MN.^10^ We therefore sought to explore whether tree‐based machine learning (ML) models could detect individuals with CH based on CBC features, through analysis of paired CBC and whole‐exome sequencing (WES) data from 431,531 United Kingdom Biobank (UKB) participants.

After excluding those with missing CBC data (*n* = 32,670), missing WES data (*n* = 36,368), or a prevalent diagnosis of a hematological malignancy (*n* = 1840), CH variant allele frequency [VAF] ≥2%) was identified in 20,860/431,531 (4.8%) UKB participants, of whom 7637 (36.6%) had large clone CH (VAF ≥10%; Figure 1A, Table S1). Using this UKB dataset, we developed a range of tree‐based models using our ML framework, which we henceforth refer to as CHIC (Clonal Hematopoiesis Inference from Counts, see Supplementary Methods).

**Figure 1:**
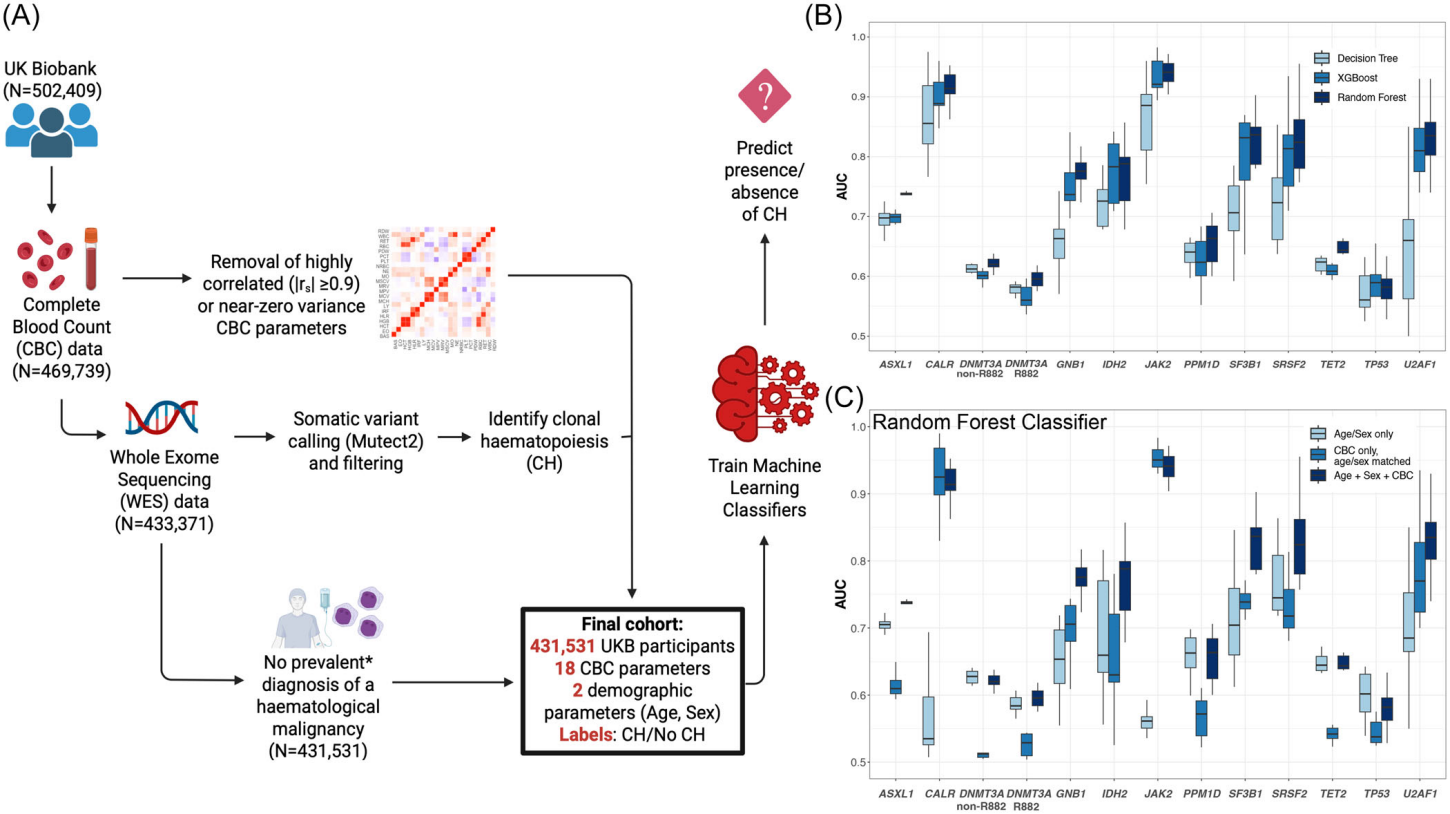
**Performance of machine learning (ML) classifiers to predict the presence of clonal hematopoiesis (CH) in the UK Biobank (UKB). (A)** Selection of the UKB cohort for training ML classifiers. Starting with participants with complete complete blood count (CBC) data (*n* = 469,739), we retained only those with whole‐exome sequencing (WES) data available (required to classify individuals as “CH” vs. “no CH”). Subsequently, we exclude individuals with a prevalent diagnosis of a hematological malignancy and those with an incident diagnosis within 30 days of recruitment, giving a final cohort of 431,531 participants. In parallel, where two CBC parameters exhibited high positive or negative correlation (Spearman, |*r*_s_ | ≥ 0.9), we retained only one of them. This image was created in BioRender. **(B)** Performance of driver gene‐specific models across all three model types (Decision Tree [DT], Random Forest [RF], and eXtreme Gradient Boosting [XGB]), using the same features. Boxplots are derived from the 10 repeats of model building; whiskers show the range of area under the receiver operating characteristic curve (AUC) values. **(C)** Performance of RF classifiers of driver gene‐specific CH, using: age and sex as the only features, or CBC features only (with age and sex matching of cases to controls, to capture the predictive performance of CBC indices alone), or age, sex, and CBC features (without age and sex matching, thereby capturing the predictive performance of CBC indices in combination with basic demographics).

Using CHIC, we initially developed binary classifiers (CH/no CH) agnostic to specific driver mutations (“any‐driver CH”), which performed modestly (median area under the receiver operating characteristic curve [AUC] 0.62–0.64, Figure S1 and Supplementary Methods/Results). Given the molecular heterogeneity of CH, we then trained driver gene‐specific classifiers and found that common mutations in epigenetic modifiers were less accurately predicted (e.g., median AUC 0.60/0.64 for *DNMT3A*/*TET2*, respectively), whereas lower prevalence, high‐risk mutations in *JAK2*, *CALR*, *SF3B1*, *SRSF2*, and *U2AF1* were more accurately predicted (median AUC 0.82–0.94, Figure 1, see also Supplementary Results, Table S2). Ensemble ML algorithms performed best (Figure 1), and further analysis showed that although age and sex alone were weak predictors, incorporating these demographic features improved classifier performance, particularly for splicing factor gene mutations (Figure 1C). As such, we focused on further optimizing Random Forest (RF) models using age, sex, and CBC indices as input features in subsequent models.

Since CH with mutations in any of *JAK2*, *CALR*, *SF3B1*, *SRSF2*, or *U2AF1* was more predictable from CBC indices and more clinically relevant (associated with higher risk of progression to MN), we next combined all five genes into a single binary classifier of CH with high‐risk genotype (CH‐HRG), to predict the presence/absence of a mutation in any of these five high‐risk genes (training on input data labeled as “CH‐HRG” vs. “no CH‐HRG”). The resulting median AUC was 0.85 on the unseen test set (Figure 2A); with performance further enhanced when predicting the presence of large (VAF ≥10%) CH‐HRG clones (median AUC on unseen test set 0.90, Figure S2). By performing iterative feature selection, we developed a compact CH‐HRG model that demonstrated stable performance when using only age and five CBC indices as input features (Figure 2B, Figure S3).

**Figure 2:**
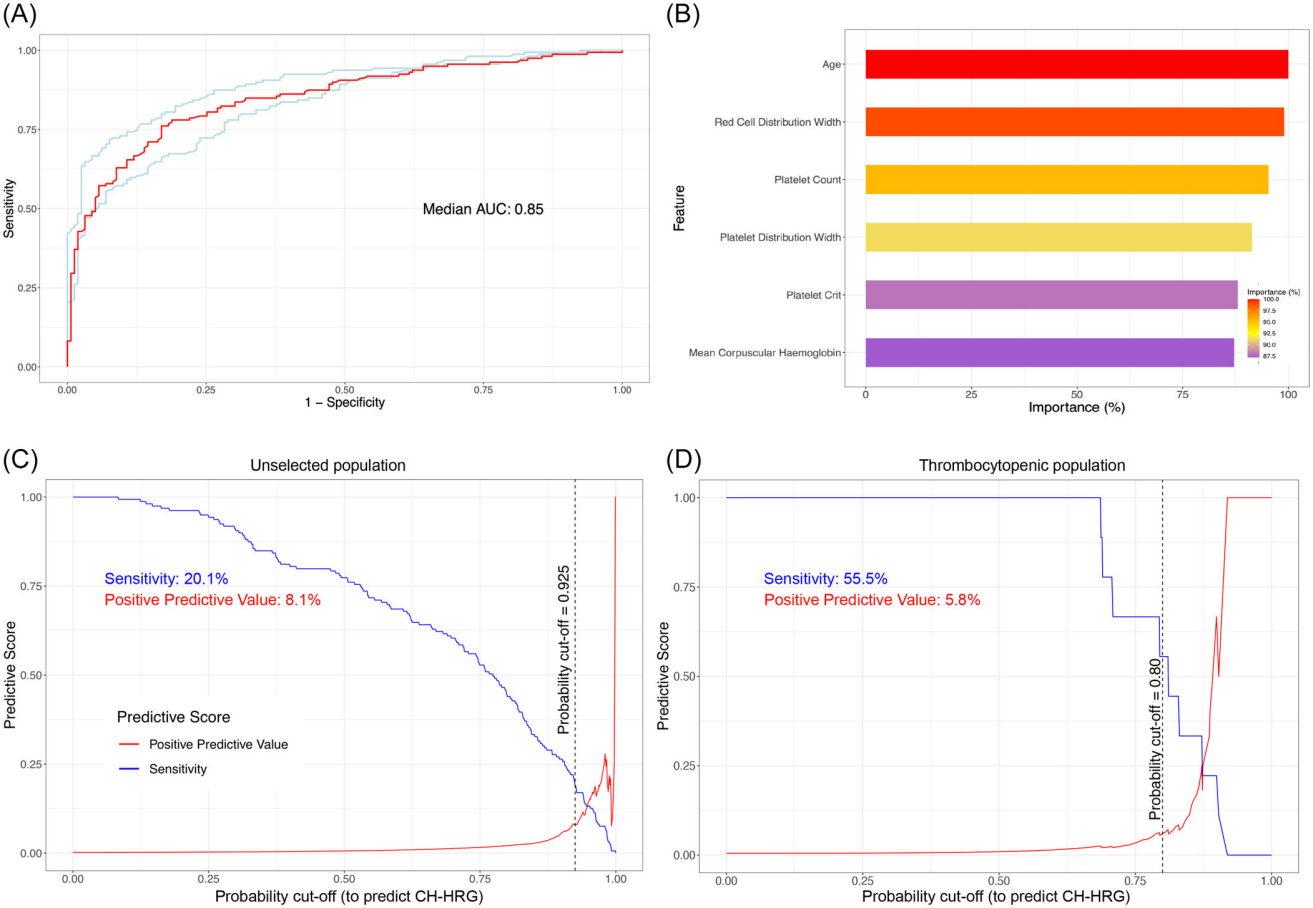
**Optimization, variable importance, and performance of a classifier of clonal hematopoiesis with high‐risk genotype (CH‐HRG). (A)** Receiver operating characteristic (ROC) curve for Random Forest model to infer the presence of CH‐HRG, based on performance on previously unseen data (median area under the ROC curve (AUC) in red, with upper and lower bounds from 10 repeats of model training in blue). **(B)** Importance, by Gini Index, of the six variables used in our compact classifier CH‐HRG. **(C)** Trade‐off between sensitivity (blue) and positive predictive value (red) for this six‐feature classifier. **(D)** Trade‐off between sensitivity (blue) and positive predictive value (red) for the same six‐feature classifier when applied to an “unseen” test set with thrombocytopenia, where CH‐HRG mutations are enriched 2.5‐fold.

Next, we assessed the optimal probability score cutoff (threshold) for our compact CH‐HRG model by examining the trade‐off between sensitivity and positive predictive value (PPV) (Figure 2C). In our UKB cohort, CH‐HRG was rare (795/431,531 UKB participants, prevalence 0.18%): since the PPV is strongly influenced by the prevalence of positive cases, this necessitated the use of a stringent probability score cutoff to minimize the number of false positives. To achieve this, we chose a cutoff probability of 0.925 (i.e., our classifier predicts the presence of CH‐HRG when the predicted probability of CH‐HRG is ≥0.925). Using this threshold resulted in a PPV of 8.1% and sensitivity of 20.1% in our unseen test cohort (*n* = 86,306), while maintaining the specificity and negative predictive value of >99.5% (Tables S3 and S4). We did not observe evidence of variation in performance of the classifier by self‐reported ancestry despite variation in the distribution of CBC indices (Supplementary Results, Figure S4, Tables S5 and S6); however, 94.1% of our final cohort described their ancestry as European.

A key limitation for the identification of CH in the UKB is the low coverage of WES, with driver genes *JAK2*, *SF3B1*, and *U2AF1* all having a median sequencing coverage of ≤31 reads,^7^ which limits our ability to identify small CH clones (e.g., with VAFs <2%). It is plausible that even small clones may be associated with CBC changes, so we examined long‐term outcomes for the 365 “false positive” cases identified by our CH‐HRG classifier and found that 38/365 (10.4%) developed MN at a median of 5.2 years from sampling. By contrast, only 317/85,782 (0.4%) of “true negatives” developed MN. Since CH is the shared precursor of the vast majority of MNs, these observations strongly suggest that a subset of “false positive” individuals had CH below the limit of detection of WES.

To further explore this hypothesis, we searched for low VAF hotspot mutations involving any of our five high‐risk genes among 38 individuals who developed MN, but were not found to have such a hotspot mutation by standard variant calling. To do so, we used “pileup” to detect mutant reads at this hotspot that were filtered out by the stringent criteria of our standard calling pipeline. This revealed that 13 of 38 apparently false‐positive UKB participants who developed incident MN had detectable CH mutations by this method, including 11 with driver mutations in *JAK2*, a low‐coverage gene. This strongly suggests that we underestimated our model performance due to the limitations of WES. In addition to cases below the limit of detection, we also examined our false‐positive cases for lower VAF mutations in a high‐risk gene and identified six cases bearing CH‐HRG mutations (two cases each of *JAK2*, *SF3B1*, and *SRSF2*‐CH) that were classified under their highest VAF mutation as *DNMT3A*, *TET2*, or *ASXL1‐*CH.

Further examination of cases identified by CHIC revealed an enrichment in cases with thrombocytosis, suggestive of undiagnosed or unannotated MPN rather than CH (Figure S5). Similarly, a few cases had cytopenias that would fall into the diagnostic criteria for clonal cytopenia of undetermined significance (CCUS) or MDS.^11^ To overcome this, we first constrained our training/test sets to individuals without cytopenias (hemoglobin <12/13 g/dL for males/females, respectively, neutrophils < 1.8 × 10^9^/L, platelets <150 × 10^9^/L), thrombocytosis (platelets > 450 × 10^9^/L), or erythrocytosis (hemoglobin >16.5/16 g/dL or hematocrit >49/48% for males/females, respectively), thereby excluding possible undiagnosed CCUS/MDS/MPN cases, and retrained our CH‐HRG classifier. This led to only a minor reduction in performance (median AUC on unseen test set 0.80, Figure S6); however, this exacerbated the trade‐off between sensitivity and PPV, leading to sensitivity and PPV of only 11.3% and 2.0%, respectively, at our proposed cutoff probability for predicting CH‐HRG of 0.875 (Figure S6D).

Next, considering the challenges posed by applying CHIC in an unselected population, we postulated that the performance may be improved if we targeted the use of CHIC to a population with abnormal CBC indices, where the prevalence of CH‐HRG is enriched. We therefore investigated the performance of CHIC on the 9576 UKB participants with thrombocytopenia (CH‐HRG was present in 53/9576, prevalence 0.45%, representing 2.5‐fold enrichment). We found that CHIC performed strongly (median AUC 0.93) in this setting, and a more lenient threshold could be applied in view of a more favorable sensitivity/PPV trade‐off (Figure 2D, Supplementary Results, Figure S7). In addition to predicting CH‐HRG, we also considered that CHIC could be used to identify the presence of high‐risk CH as determined by recently developed MN risk prediction tools.^7^, ^8^ By training a classifier using labels based on risk score (10‐year MN risk ≥10%) rather than genotype, we could robustly differentiate between high‐risk CH and controls (median AUC 0.96, see Supplementary Results, Figure S8), but as these risk stratification tools were trained on UKB blood count data, this strong performance may arise from overfitting and requires validation in an independent cohort.

We developed an ML framework and assessed an RF classifier that predicts the presence of CH‐HRG from just five CBC variables and the individual's age. This approach, named CHIC, can discriminate between individuals with and without mutations in five CH genes associated with high risk of developing MN. Notably, CHIC retained the ability to discriminate high‐risk CH cases from controls even among individuals without cytopenias, erythrocytosis, or thrombocytosis, suggesting that it can highlight individuals that may not otherwise come to medical attention. CHIC is an important first step towards developing a scalable screening test to identify individuals likely to harbor high‐risk CH, who would then be prioritized for targeted NGS. Clinically, this could be utilized to reduce the number needed to sequence (NNS) to identify one case of CH‐HRG, thus making screening at scale more feasible and justifying the need to perform genetic testing. Even with its current limitations, the use of CHIC with a stringent cutoff probability on individuals without cytopenia or thrombo‐/erythrocytosis would still markedly reduce the NNS from 727 to 40 individuals per case of high‐risk CH (based on the prevalence of high‐risk CH in an unselected population vs. in those predicted to have high‐risk CH by CHIC). Also, when we applied CHIC without constraints on CBC indices, it identified individuals with high‐risk mutations and indices consistent with CCUS/MDS or MPN, rather than CH, suggesting it could also be utilized to identify undiagnosed individuals without relying on clinician recognition and referral.

However, despite its promising metrics, the performance of CHIC in an unselected population was limited by the rarity of high‐risk CH, necessitating the ceding of sensitivity to achieve an acceptable PPV. One approach for enhancing the performance of CHIC is to target its use to a population with a higher prevalence of high‐risk CH. For example, targeting CHIC to a thrombocytopenic cohort substantially ameliorated the trade‐off between sensitivity and PPV. We anticipate that CHIC will generalize to specific contexts where mutations in *JAK2*, *CALR*, *SF3B1*, *SRSF2*, and *U2AF1* predominate (e.g., thrombo/erythrocytosis or cytopenias), although in other “high‐risk” contexts, such as detecting clonal expansions post‐chemotherapy, CHIC may not generalize well since both the mutational (*TP53* and *PPM1D*‐enriched) and CBC (therapy‐related CBC perturbations) landscape differ substantially from the context in which CHIC was trained and optimized. We expect that CHIC would be best applied to community‐dwelling adults attending primary care, since an inpatient population would be expected to have higher rates of inflammation and infection that could perturb CBC indices and detrimentally affect model performance.

An alternative approach to improve performance would be to integrate higher resolution CBC data into the CHIC classifier, since the most discriminative CBC indices for high‐risk CH are derived summary statistics calculated from single‐cell measurements (e.g., RDW, platelet disribution width, and mean cell hemoglobin). The use of embeddings of raw single‐cell measurements has the potential to improve the prediction of high‐risk CH, for example, by revealing the presence of a bimodal distribution in cell size distribution arising from a clonal population of cells with distinct indices or identifying other characteristic patterns of variation in these measurements. Such raw (or “non‐classical”) CBC traits have recently been exploited to explore genetic associations with blood cell morphology.^12^ By retrofitting CH‐HRG screening onto a routine blood test, we believe our CHIC approach presents an important step towards scalable, practical, and inexpensive ML‐based screening for CH‐HRG and provides a proof‐of‐concept that individuals with CH‐HRG can be differentiated from those without, based on CBC indices.

## AUTHOR CONTRIBUTIONS

**William G. Dunn**: Writing—review and editing; writing—original draft; investigation; methodology; visualization; software; formal analysis. **Isabella Withnell**: Investigation; methodology; writing—review and editing; formal analysis; software. **Muxin Gu**: Methodology. **Pedro Quiros**: Methodology. **Sruthi Cheloor Kovilakam**: Methodology. **Ludovica Marando**: Methodology. **Sean Wen**: Methodology. **Margarete A. Fabre**: Methodology. **Irina Mohorianu**: Methodology; supervision; writing—review and editing. **Dragana Vuckovic**: Writing—review and editing; supervision; methodology; conceptualization. **George S. Vassiliou**: Conceptualization; writing—review and editing; supervision.

## CONFLICT OF INTEREST STATEMENT

G.S.V. is a consultant to STRM.BIO and holds a research grant from AstraZeneca for research unrelated to that presented here. S.W. is an employee of AstraZeneca. M.A.F. is an employee and stockholder of AstraZeneca. The other authors declare no competing interests.

The data that support the findings of this study are openly available in the UK Biobank at https://www.ukbiobank.ac.uk, reference number 56844 and 69328.

## FUNDING

W.G.D. is funded by a Clinical Research Fellowship from Cancer Research UK (CTRQQR‐2021\100012). G.S.V. receives funding from a Specialist Centre of Research grant from the Leukemia and Lymphoma Society (7035‐24); he also holds a Cancer Research UK Senior Cancer Fellowship (C22324/A23015), and work in his laboratory is also funded by the Kay Kendall Leukemia Fund, Astrazeneca, Blood Cancer UK, and the Wellcome Trust.

## Supporting information

Supporting Information.

## Data Availability

All data used in this study are publicly available from the UK Biobank (https://www.ukbiobank.ac.uk/). Researchers may apply for access to the UK Biobank data via the Access Management System (https://www.ukbiobank.ac.uk/enable-your-research/apply-for-access). *Code availability*. Scripts used to query the UK Biobank dataset are available from: https://github.com/IsabellaWithnell/Predicting_CH. Scripts used to implement the machine learning framework described in the manuscript are available from: https://github.com/billydunn/chic.
