# Supplementary material for: CHIC: A machine learning framework for inferring the presence of high‐risk clonal hematopoiesis using complete blood count data from 431,531 UK Biobank participants
Source: Hemasphere. 2025 Jul 3;9(7):e70169. doi: 10.1002/hem3.70169 (PMC12226720; doi:10.1002/hem3.70169)
Supplement: Supplementary file 1 — Supporting Information. [file HEM3-9-e70169-s001.docx]

**Supplementary Materials**

**Table of Contents**

Supplementary Methods Page 2-4

Supplementary Results Page 5-8

Supplementary Figures Pages 9-21

Supplementary Tables Pages 22-31

**Supplementary Methods**

**Study design and participants**

We utilised data from the UKB (<https://www.ukbiobank.ac.uk/>), a population-based cohort of 502,536 volunteers from the United Kingdom recruited between 2006-2010 when aged between 37 and 73 years and followed up since^1^. Participants’ data were accessed under approved UKB applications number 56844 and 69328.

To derive a dataset for use in our ML pipeline, we excluded UKB participants with any missing CBC variables and those without WES data. Since CH is defined by the presence of a leukaemia-associated somatic driver mutation in an individual without an apparent blood neoplasm, participants with a previous diagnosis of a haematological malignancy were excluded from the final dataset, as were those who developed an incident haematological malignancy within 30 days of recruitment to the UKB. After exclusions, 431,531 participants were retained for downstream analyses.

**Identification of clonal haematopoiesis from whole exome sequencing data**

To identify UK Biobank participants with CH driver mutations, we used mutation calls derived from blood WES data, previously published by Gu et al^2^. Briefly, Mutect2 was run in tumour-only mode against a panel of 38 CH-associated genes. Germline variants were filtered using a panel of normals. Putative somatic variants were further filtered using FilterMutectCalls, while variants flagged as “germline” were rescued if present at least five times in the set of putative somatic variants. To derive a final list of driver mutations, variants were firstly filtered based on the number of alternate reads (≥2), presence on forward and reverse strands, minimum read depth (≥7/≥10 for SNVs/indels respectively) and minor allele frequency (<0.001) in gnomAD^3^. The criteria described by Vlasschaert et al.^4^ were then used to further filter germline variants and sequencing artefacts. To overcome previously reported issues with mapping at the *U2AF1* locus^5^, *U2AF1* mutations were called separately using Samtools^6^ mpileup to identify single nucleotide variants (SNVs) at known hotspots, with variants supported by ≥3 reads and at VAF >0.1 retained for downstream analyses. UKB participants were subsequently labelled as “any-driver-CH” or “no CH” based on the presence or absence of a driver mutation(s) at VAF ≥2%. For input to gene-specific models of CH, we additionally labelled UKB participants by driver gene (e.g. “*TET2*-CH”, “*SRSF2*-CH”, etc vs “no CH”). Individuals with ≥2 driver mutations were labelled on the gene with the highest VAF.

**Variable selection**

We extracted all 22 CBC variables available in the UKB and augmented the feature set with the participants’ age and sex. Some CBC variables are closely related or derived from one another; to assess collinearity we computed a pairwise Spearman’s rank correlation coefficient (r_s_) and excluded variables with a |r_s_| ≥0.9. This led us to exclude haematocrit, high light scatter reticulocyte count and the total white blood cell count, whilst retaining their highly correlated counterpart features: haemoglobin concentration, reticulocyte count and neutrophil count, respectively. Nucleated red blood cell count (NRBC) was also excluded as it exhibited near-zero variance (106 unique values, NRBC=0 in 98.9% of UKB participants).

**Supervised machine learning model development**

Having derived ground truth labels from WES data, ML models were built for “any-driver-CH” (variant allele frequency, VAF, ≥2% with a driver mutation in any CH gene), “large clone any-driver-CH” (as previous but VAF ≥10%), and each individual driver gene CH subtype.

To develop a binary classifier for predicting the presence/absence of CH, we trained and evaluated a selection of tree-based machine learning models: Decision Trees, Random Forest and Extreme Gradient Boosting (XGBoost) Trees. Tree-based approaches were preferred since the set of input features was heterogeneous (continuous and categorical); moreover, these models, augmented with statistical analyses, may also capture the interaction between features. Aside from the assessment of near-zero variance and collinearity, no further pre-processing was applied to the input dataset.

All of the 18 CBC parameters selected were used as features, in addition to basic demographic data (age at sampling and sex). Since the UKB CH dataset was imbalanced, with significantly more controls (no CH) than cases (CH), random down-sampling was performed to achieve a 1:1 ratio of cases:controls in the input data, to enhance model training and convergence; this down-sampling process was repeated ten times iteratively (Supplementary Figure 9). Subsequently, down-sampled datasets were partitioned on 80:20 training:test ratio. All models were built using ten repeats of ten-fold cross-validation setups; a grid-search approach was used to tune the relevant hyperparameters (Supplementary Table 6). To avoid technical bias from the down-sampling step, a modified cross-validation approach was adopted, training and evaluating each ML model ten times iteratively, each time using a different random down-sample of the majority (control) class, thereby quantifying the robustness and stability of each model to variation in the subset of control samples or train/test partition (Supplementary Figure 9). Model performance was assessed on the unseen test data, on receiver operating characteristic (ROC) curves and area under the curve (AUC), in addition to sensitivity and specificity.

From the Random Forest models, we determined variable importance by computing the mean decrease in node impurity from splitting on each feature (measured by Gini index), averaged across all trees and across each of the ten repeats of model-building, using the importance() function from the randomForest package in R (v4.7.1)^7^. The consistency across top-ranked variables per driver was visualised using quantitative Venn diagrams (upset plots, ComplexUpset package) on the top two variables. The feature selection was performed by ranking all *n* features by importance, in descending order, and iteratively excluding the least informative feature, to determine a minimum set of highly predictive features.

To assess the scalability of the final model in a “real-life” setting, i.e. with class imbalance, we added unseen control subjects (no CH) to the test set to match the prevalence of CH cases to that in the entire UKB cohort. We examined the trade-off of sensitivity (which is independent of prevalence), positive predictive value (which is dependent on prevalence) and the model prediction score, using this to determine the optimal cut-off score, that minimises the false positives whilst retaining adequate sensitivity.

All ML models were built using the Caret v6.0.91 package in R v3.6.3^8^. All code used to implement our ML framework is publicly available on GitHub: <https://github.com/billydunn/chic>.

**Developing a model of CH-HRG with “normal” CBC indices**

To investigate whether our models could detect individuals with CHIP, who, by definition, have relatively normal CBC indices, we further constrained our training and test cohorts to include only UKB participants who did not have a cytopenia (haemoglobin <12/13 g/dL for males/females respectively, neutrophils <1.8 x 10^9^/L, platelets <150 x10^9^/L), erythrocytosis (haemoglobin >16.5/16 g/dL or haematocrit percentage >49/48% for males/females respectively) or thrombocytosis (platelets > 450 x 10^9^/L). These thresholds were derived from the definitions of cytopenias, erythrocytosis and thrombocytosis used in the diagnostic criteria for CCUS, MDS and MPN in the 5^th^ edition of the World Health Organisation Classification of Haematolymphoid Tumours^9^. We then used this constrained population as input to our ML model training & validation pipeline, as outlined in our Methods section in the main manuscript. We only applied these thresholds to the model of “normal” indices, to compare performance with the classifier trained on the UKB dataset without CBC restrictions.

**Supplementary Results**

**Evaluating the effect of model type, class labels and input features on classifier performance**

We firstly examined whether CH could be predicted from CBC data in the UKB using models agnostic to individual gene driver mutations (henceforth “any-driver CH”). Using CHIC, we generated binary classifiers (CH/no CH) of “any-driver CH” using tree-models with 18 CBC variables plus age and sex as features. Classifiers of “any-driver CH” were better than random classification, but with modest performance across all model types (median AUC on unseen test set 0.62, 0.64 and 0.62 for DT, RF and XGB models respectively) (Figure 1B).

CH is a molecularly heterogenous entity, and we posited that the nature and strength of the CBC phenotype conferred by a somatic mutation may vary according to the specific driver gene. We therefore trained driver gene-specific binary classifiers (with labels “driver gene CH”/“no CH”) using the same input variables as for the “any-driver” CH models. The most prevalent forms of CH, driven by mutations in *DNMT3A* and *TET2*, were not robustly detectable; this conclusion held for *DNMT3A*-R882 hotspot mutations, which are associated with a higher risk of transformation to AML^2^ (median AUC 0.60, 0.62 and 0.64 for *DNMT3A*-R882, *DNMT3A*-non R882 and *TET2* RF models respectively) (Figure 1C). By contrast, CH driven by lower prevalence but higher risk driver mutations in the genes *JAK2*, *CALR*, *SF3B1*, *SRSF2* and *U2AF1* performed much better (median AUC 0.94, 0.91, 0.84, 0.82, 0.84 respectively for RF models) (Figure 1C). Since Random Forest (RF) models generally exhibited the best performance across the driver genes (Figure 1C, Supplementary Table 2), we focused on further developing and exploring RF models.

CH is strongly associated with age, whilst some driver genes exhibit sex bias. As such, we sought to quantify the influence of age and sex in our RF models. To understand the influence of age and sex in the RF models, we trained each set of driver gene-specific RF models in three iterations: i) with age and sex as the only features, ii) with CBC indices as the features, whilst age- and sex-matching cases to controls (to capture the predictive performance of CBC alone), and iii) with age, sex and CBC indices as features, without age- and sex-matching of cases/controls (to capture the predictive performance of both basic demographics and CBC indices). The performance of models trained with only age and sex as features was generally poor (median AUC <0.75 in all cases, Figure 1D); an exception was the age/sex-only model of *SRSF2-*CH, in line with the sharp rise in prevalence of *SRSF2*-CH with advancing age and its strong association with male sex^10^. Classifiers of CH driven by high-risk genes *JAK2*, *CALR*, *SF3B1*, *SRSF2* and *U2AF1* performed best (median AUC of 0.94, 0.91, 0.84, 0.82 and 0.84 respectively) when using CBC indices as features and age/sex matching cases to controls in the training and test sets. The predictability of the presence of CH driven by mutations in splicing factor genes (*SF3B1*, *SRSF2* and *U2AF1*) was augmented when age and sex were added as features and age-/sex-matching was omitted. Acknowledging the predictive power of age and sex, we added these features to CBC indices in subsequent models.

**Classifiers of high-risk clonal haematopoiesis as determined using MN-predict**

In light of the development of risk-stratification tools to estimate the risk of progression from CH to MN, we next explored using CHIC to train a classifier based on the risk of incident MN. Of our 795 participants with CH-HRG, most would have been designated as high (n=100) or intermediate (n=318) risk by Clonal Hematopoiesis Risk Score (CHRS, Supplementary Table 7); importantly, CHRS does not consider *U2AF1* mutations, meaning that most of these mutations (39/53) would be misclassified as low-risk based on CHRS.

Importantly, both the CHRS and MN-predict risk-stratification tools were developed using the UK Biobank, and both integrate CBC variables into their risk predictions. As such, there is a risk that developing a classifier using CBC parameters as input features with labels based on predicted risk becomes a tautological problem, since individuals with predicted higher risk tend to have perturbed indices, and the classifier is then trained to predict class labels based on such indices. Considering these concerns, we chose to assign class labels using predictions from MN-predict, since this i) integrates CBC indices as continuous variables (whereas CHRS used binarised indices), ii) considers *U2AF1* mutations, and iii) was developed by our team, allowing us to identify and exclude the portion of the UK Biobank used in the training cohort (n=207,036), thereby minimising overfitting. We assigned class labels (high-risk CH/no high-risk CH) based on a predicted risk of any MN of ≥10%, and used CHIC to train a Random Forest classifier of high-risk CH based on these labels. This performed very strongly (median AUC 0.96, Supplementary Figure 8A), but since high-risk CH as defined by MN-predict was even rarer than CH-HRG, this again did not scale well (Supplementary Figure 8B). In view of our concerns about overfitting, we would urge caution in interpreting the performance of the model in this classification task and suggest that its use to predict high-risk CH would require validation in an external dataset.

**The addition of biochemical variables does not improve performance**

Though our primary objective was to develop a classifier that could infer the presence of CH from CBC indices, we also considered whether the addition of any biochemical tests would improve the prediction of CH-HRG. As such, we used CHIC to train a Random Forest classifier with age, sex, CBC indices and biochemical variables (total bilirubin, alanine transaminase, aspartate transaminase, alkaline phosphatase, cholesterol, creatinine, glycated haemoglobin, urate, insulin-like growth factor-1 levels) as input features. Using these additional input features, we found the performance of the classifier of CH-HRG was unchanged (median AUC with and without biochemistry 0.86 vs 0.85 respectively, Supplementary Figure 11).

**Performance of the classifier in the context of thrombocytopenia**

Given the issues identified in scaling the classifier to an unselected population, we considered that a useful application of CHIC would be to differentiate those with abnormal blood indices bearing high-risk mutations from those without. To examine this in further detail, we used CHIC to train Random Forest classifiers, training only on the subset of the UK Biobank with thrombocytopenia (n=9576), and labelling the positive class based on the presence of CH-HRG (that is, *JAK2*, *CALR*, *SF3B1*, *SRSF2* or *U2AF1*) or only on splicing factor mutations (*SF3B1*, *SRSF2*, *U2AF1*), which are more prevalent MPN-associated mutations in this setting. We compared the performance of these “bespoke” classifiers, trained in the setting of thrombocytopenia, with our classifier of CH-HRG that was trained without constrains on CBC indices. Surprisingly, the bespoke classifiers that were trained only in the context of thrombocytopenia exhibited marginally worse performance (median AUC 0.89 and 0.87 for CH-HRG and splicing-CH classifiers respectively) than the classifier of CH-HRG trained on the unselected cohort (Supplementary Figure 7). The superior performance of the classifier trained on the unselected cohort likely stems from its exposure to a larger and more diverse training set, enabling it to better approximate the underlying distribution of features associated with the positive class (the presence of CH-HRG).

**Classifier performance in European versus non-European ancestry**

We grouped UK Biobank participants by self-reported ancestry into European (n=407,057, 94.3%), African (n=2833, 0.6%), Asian (n = 9619, 2.2%) or mixed/unknown ancestry (n=12,022, 2.8%). We observed significant variation in CBC indices by self-reported ancestry, including expected associations, such as a lower neutrophil count in individuals with African ancestry (Supplementary Figure 4). Despite this, we did not observe evidence of a variation in the performance of the classifier of CH-HRG between those with European or non-European ancestry, though the numbers in the predicted positive classes were small, precluding formal statistical testing (Supplementary Tables 5 & 6).

**Assigning ground truth labels based on VAF gene mutations does not impact performance**

In our supervised ML pipeline, where individuals harboured ≥2 driver mutations, we assigned gene-specific class labels based on the largest clone, reasoning that the larger clone might be expected to have the dominant effect on CBC indices. We considered that this approach may cause individuals with subclonal mutations in high risk genes to be misclassified in our model of CH-HRG. We therefore re-assigned class labels based on the presence of *any* mutation in a high-risk gene, even if subclonal to another driver. This increased the number of participants identified as harbouring CH-HRG by 8%, from 795 to 859 cases, driven largely by subclonal *SRSF2* mutations (Supplementary Table 8). We then re-trained the classifier using our CHIC framework, and found no appreciable difference in performance (median AUC training with any driver versus largest clone 0.86 vs 0.85 respectively, Supplementary Figure 10).

**Variable importance highlights blood count associations with driver mutations**

In addition to their use for prediction, we considered that CHIC ML models could also uncover novel associations between individual driver mutations and CBC indices. By evaluating variable importance across all the driver-gene-specific classifiers, and summarising the overlap between the top two features in each model (Supplementary Figure 12A), we observed known or expected associations: age was highly predictive across models, *JAK2*-CH and *CALR*-CH shared platelet count and plateletcrit as important features whilst MCV was predictive of *SF3B1*-CH.

We also observed unexpected associations: for example, the basophil count was discriminative for predicting the presence *GNB1*-CH only, whilst eosinophil count was discriminative for the presence of *IDH2*-CH. Examining the distribution of each of these CBC variables in the UKB, we found that individuals with *GNB1*-CH had a significantly increased basophil count (p = 5.93 x 10^-11^, Wilcoxon Rank Sum test), with a 4.5-fold increase in the prevalence of basophilia >0.1 x 10^9^/L and 8.6-fold increase in the prevalence of basophilia >0.2 x 10^9^/L, relative to participants without a *GNB1* driver mutation (13.8% vs 3.2% and 5.0% vs 0.6% for basophilia >0.1 and >0.2 x 10^9^/L, n = 178/431,353 for *GNB1* mutant/wild-type respectively) (Supplementary Figure 12B). Individuals with *IDH2*-CH had significantly lower eosinophil counts (p = 3.63 x 10^-10^, Wilcoxon Rank Sum test) and a propensity to eosinopenia, with 12/92 (13.0%) participants with *IDH2*-CH having absolute eosinopenia (eosinophils = 0 x 10^9^/L ) and 45/92 (48.9%) having an eosinophil count <0.1 x 10^9^/L. By contrast, individuals without *IDH2* mutations had rates of eosinopenia of 2.9%/20.8% for absolute/<0.1 eosinopenia respectively (n = 70/431,461 for *IDH2* mutant/wild-type respectively) (Supplementary Figure 12C). *IDH2*-CH demonstrated a significant association between eosinophil count and clone size (r_s_ = -0.51, p = 2.67 x 10^-7^), but we observed no such association between *GNB1*-CH and basophil count (r_s_ = 0.13, p = 0.09) (Supplementary Figure 13). However, as basophils are the rarest of the white blood cells, their counts are zero-biased, which may have limited our ability to detect a correlation with clone size. Of note, eosinophil counts are also zero-biased, and given the skewed distributions and small numbers of cases, these observations may be sporadic and require validation in independent cohorts.

**Supplementary Figures**

**Supplementary Figure 1: Performance of classifiers of any-driver CH.** The ROC curves of the performance of three types of machine learning classifier are shown. Class labels used in training are “CH” or “No CH”, but do not distinguish between different types of driver mutation. The ROC curves displayed are those approximating median AUC (area under the ROC curve) from ten repeats of model training. DT = Decision Tree, RF = Random Forest, XGB = eXtreme Gradient Boosting.

**

***Supplementary Figure 2: ROC curve for model of large clone CH-HRG.*** *Large clone CH-HRG was defined as CH driven by JAK2, CALR, SF3B1, SRSF2 or U2AF1, with a VAF of ≥10%. This model utilises all 18 CBC variables, age and sex as input features. The ROC curve has been constructed and AUC calculated based on performance in the unseen test set. Red, performance of model approximating the median AUC. Upper and lower bounds represent performance of the models with the maximum and minimum AUC from ten repeats of model training respectively.*

**Supplementary Figure 3:** **Performance of the classifier of CH-HRG versus number of input features.** Here, the classifier has been re-trained iteratively, each time excluding the least important feature as measured by Gini index). The performance of the classifier is unchanged with only six input features.

**Supplementary Figure 4: Variation in complete blood count indices by self-reported ancestry.** Cells are coloured by the difference in median CBC values, scaled with respect to the interquartile range. Differences are calculated relative to the median value amongst those with self-reported European ancestry.

**

***Supplementary Figure 5:*** ***Distribution of CBC indices in high-risk cases in the test cohort.*** *Red = predicted as a case (true positive), blue = predicted as control (false negative), using our proposed stringent cutoff for our six-feature model (input features age, RDW, PLT, PDW, PCT, MCH). Most true positives have high platelet counts, which may be consistent with an unannotated MPN.*


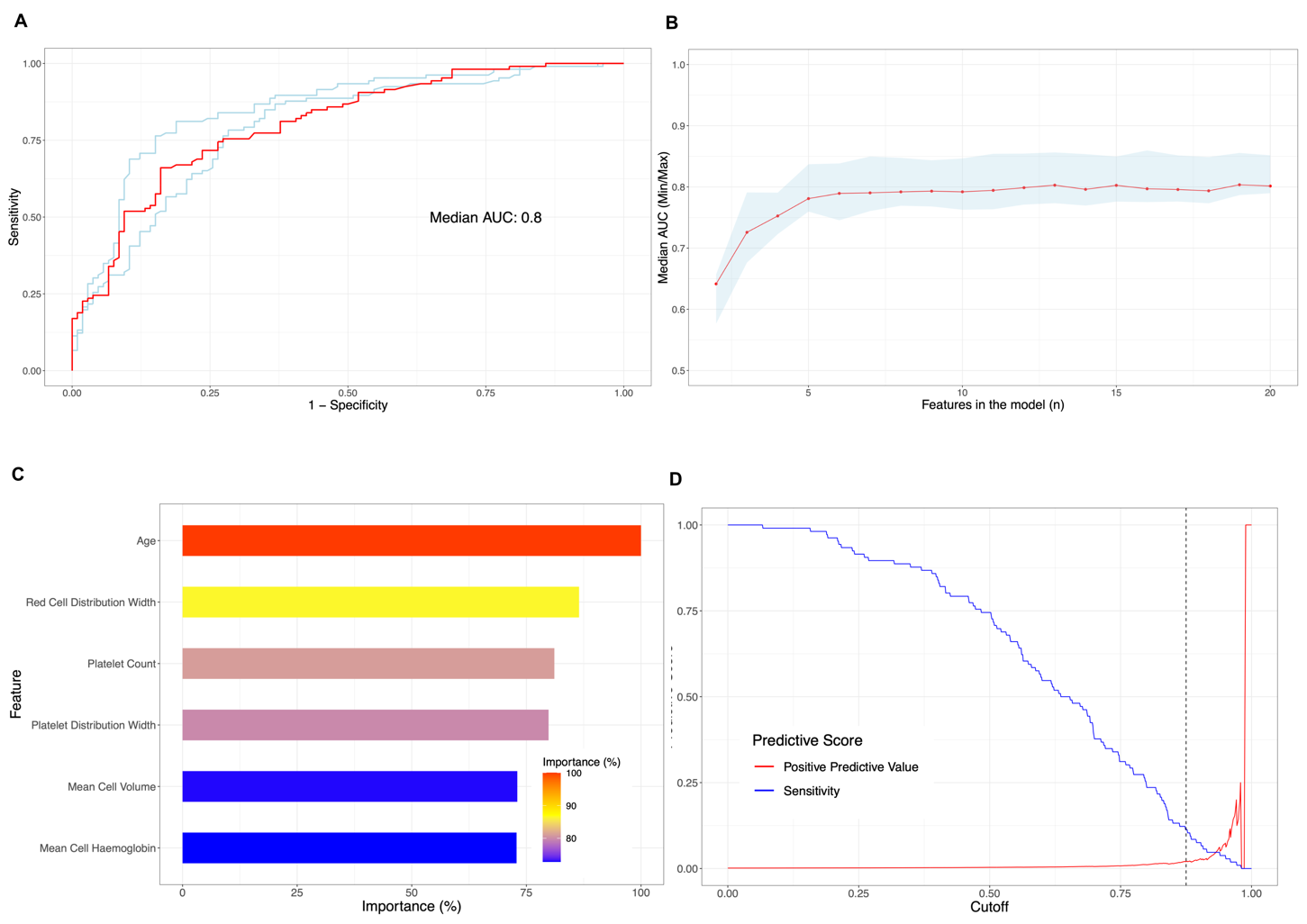


***Supplementary Figure 6: A classifier of CH-HRG with normal CBC indices.*** *Here, the model has been trained and tested on UK Biobank participants without cytopenias or thrombo-/erythrocytosis. (A) ROC curve for the optimised Random Forest model and AUC calculated based on performance in the unseen test set. Red, performance of model approximating the median AUC. Maximal Upper and lower bounds represent performance of the models with the maximum and minimum AUC from ten repeats of model training respectively. (B) Summary of impact of iterative feature selection on model performance (by AUC), demonstrating that performance is stable with a selection of six input features. (C) variable importance summary (by Gini Index, scaled to the most important variable) of features in our six-feature classifier. (D) summary of trade-off between sensitivity (blue) and positive predictive value (red) for this six-feature classifier.*

**

**Supplementary Figure 7: Performance of classifiers in individuals with thrombocytopenia.** Here, 3 classifiers are applied only to the unseen test cases where the platelet count is <150 x10^9^/L. The classifier of CH-HRG, which was trained on an unselected UK Biobank cohort (that is, no constraints were placed on CBC indices) exhibits the best performance by AUC (red, median AUC: 0.93). By contrast, when “bespoke” training was performed on only the subset of UK Biobank participants with thrombocytopenia to detect any CH-HRG mutations (JAK2, CALR, SF3B1, SRSF2, U2AF1), performance was marginally worse. When training was further refined to only detect splicing factor mutations (SF3B1, SRSF2, U2AF1), which are the predominant high-risk gene mutations in this context, performance decreased further.

**

**Supplementary Figure 8: Performance of a classifier of high-risk CH.** Here, high-risk CH has been determined using MN-predict, and defined as a predicted ten-year risk of any MN of ≥10%. Panel A: The classifier shows strong performance in discriminating between positive (high-risk CH) and negative (no high-risk CH) classes. Panel B: Despite this strong performance, as high-risk CH is rare, the sensitivity-PPV trade-off still limits the scalability of the classifier in an unselected population.


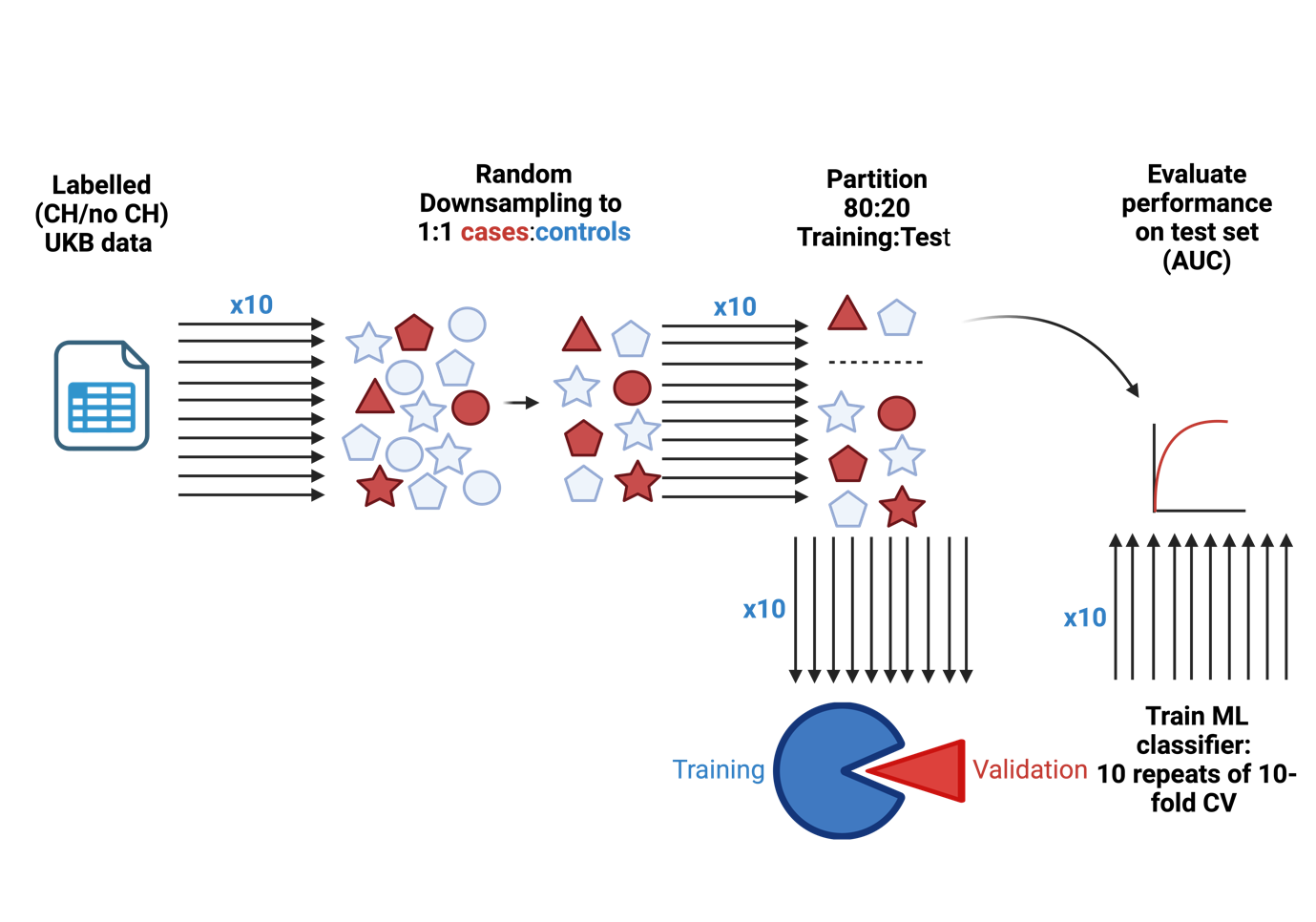


***Supplementary Figure 9: Overview of the machine learning framework, CHIC.*** *The input comprises our master dataset of 431,531 UKB participants with variables age, sex and CBC parameters, each labelled as “CH” or “no CH” (or for gene-specific models, gene-specific CH or no-CH). To enhance model training and convergence, we randomly down-sample from the master dataset ten times, to produce ten subsamples each with a 1:1 ratio of cases (CH, red) to controls (no CH, blue). Each dataset has the same set of cases, but a random sample of controls. We then partition the ten datasets in an 80:20 ratio corresponding to training:test cohorts, and train ten ML models, each time using ten repeats of ten-fold cross-validation to control for overfitting. Grid search was used to tune the relevant hyperparameters for each model type. The performance of each model was evaluated on the unseen test set, using area under the ROC curve (AUC) as the primary performance measure. By generating the starting dataset ten times, and independently partitioning each dataset and training/evaluating each model, we assess the robustness and stability of each model to variations in the train/test split and in the random sampling of controls.*

**

**Supplementary Figure 10: Impact of assigning class labels based on largest clone.** The figure shows the ROC curves for classifiers of CH-HRG when trained on class labels assigned based on the largest clone (blue) or based on the presence of any high risk gene mutation (JAK2, CALR, SF3B1, SRSF2, U2AF1) irrespective of whether there is another driver present at higher variant allele frequency.

**Supplementary Figure 11:** **Impact of addition of biochemical variables on classifier performance.** The figure shows ROC curves of classifiers of CH-HRG with (red) and without (green) addition of biochemical variables (total bilirubin, alanine transaminase, aspartate transaminase, alkaline phosphatase, cholesterol, creatinine, glycated haemoglobin, urate, insulin-like growth factor-1 levels).


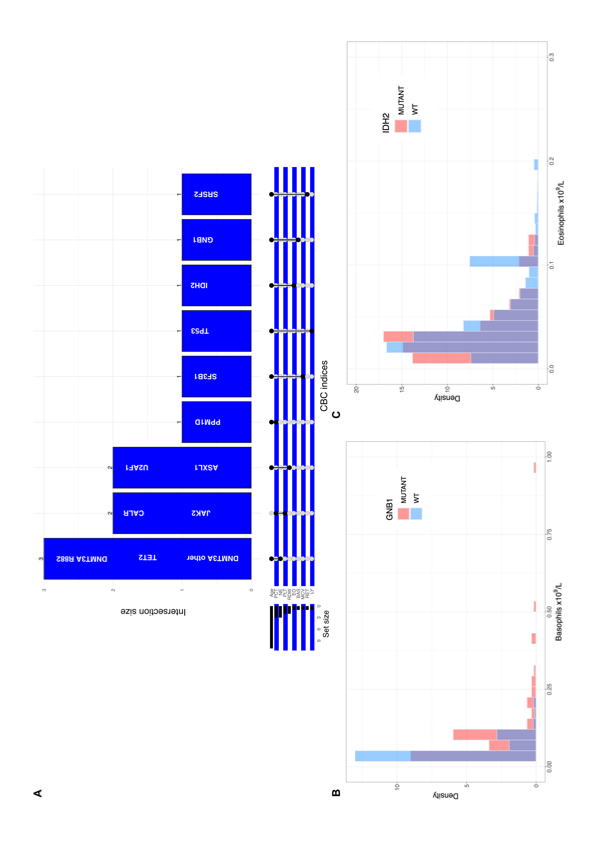

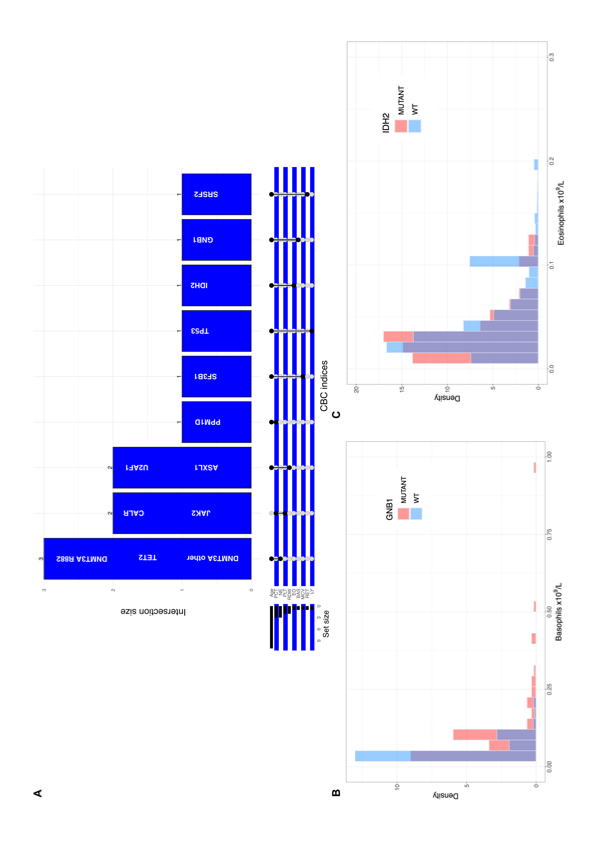


*
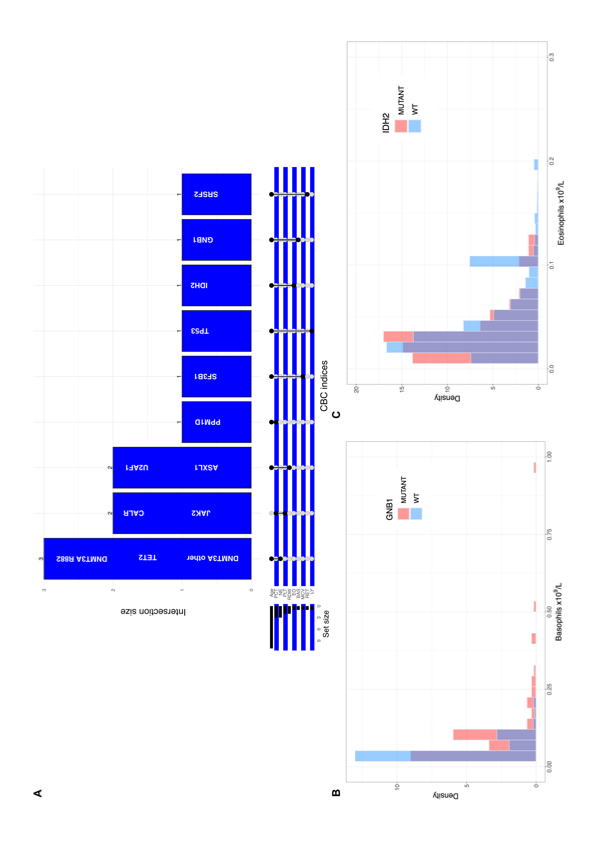
*

***Supplementary Figure 12:*** ***Machine learning models of driver gene CH for biological inference.*** *Panel A shows an Upset plot generated by computing variable importance and summarising the overlap (vertical bars) between the top two most important variables in RF classifiers of driver gene CH. This captures expected associations (JAK2 and CALR share platelet crit and platelet count as their top two variables), but also unveils unexpected associations, such as the importance of basophil count for predicting the presence of GNB1-CH and the importance of eosinophil count in predicting the presence of IDH2-CH. PCT = platelet crit, NE = neutrophil count, PLT = platelet count, RDW = red cell distribution width, EO = eosinophil count, BAS = basophil count, MCV = mean cell volume, RET = reticulocyte count, LY = lymphocyte count. Panel B shows a histogram of basophil counts in carriers of GNB1-CH (n = 178) versus those without (n = 431,353); the basophil count is shifted to the right in those with GNB1-CH, who have a relatively high prevalence of basophilia. Panel C shows the histogram of eosinophil counts in individuals with (n = 70) and without (n = 431,461) IDH2-CH; there is a higher proportion of absolute eosinopenia (i.e. eosinophil count = 0) in individuals with IDH2-CH. In both panels B and C, the y axis is density, to facilitate direct comparison between imbalanced classes.*

*
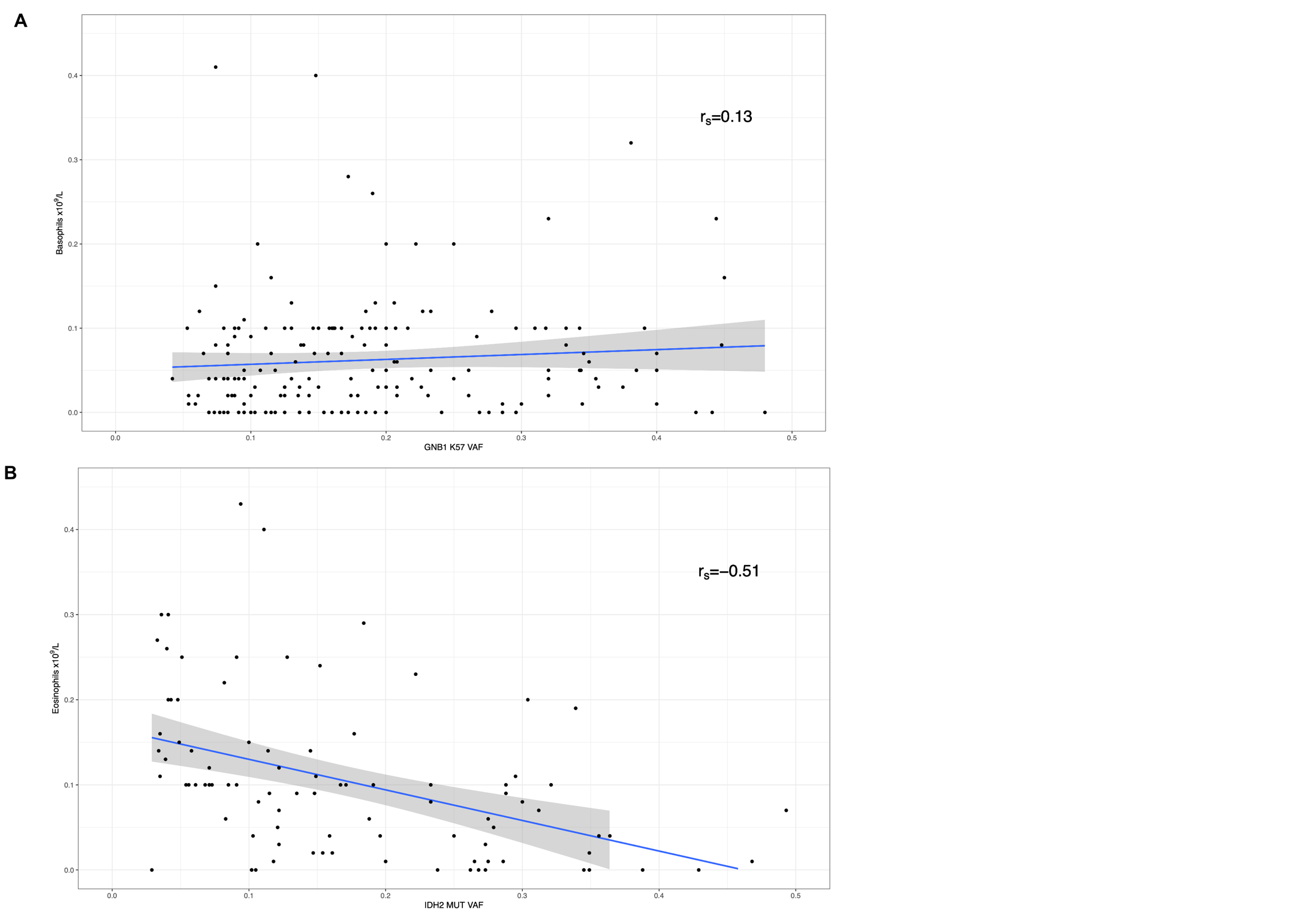
*

***Supplementary Figure 13:*** ***Correlation between clone size and indices.*** *Panels A and B show the correlation between basophil/eosinophil count and GNB1/IDH2 mutation variant allele fraction (VAF), respectively. R_s_ denotes Spearman’s rho value. IDH2 clone size exhibits a significant inverse correlation with eosinophil count (Panel B, p = 2.67 x 10^-7^), but we observed no significant correlation (p = 0.09) between GNB1 clone size and basophil count (Panel A).*

**Supplementary Tables**

***Supplementary Table 1: Clonal haematopoiesis proportions in the filtered cohort (n = 431,531).***

**

***Supplementary Table 2:*** ***Performance across model types and driver genes.*** *DT, RF and XGB denote Decision Tree, Random Forest and eXtreme Gradient Boosting models respectively. Included here are additional gene-specific models that were constructed but not included in the main manuscript, which focuses on the most common or highest risk CH drivers.*

**

**Supplementary Table 3: Performance of the classifier of CH-HRG**. Confusion matrix showing predicted class vs ground truth for cases in the unseen test set. The classifier of CH-HRG has been used to generate predictions in this unseen dataset using a stringent probability cut-off (0.925) to minimise false positives.

**

***Supplementary Table 4: Breakdown of true positives by driver gene.*** *Detection of specific driver genes by combined classifier of CH-HRG, using stringent cutoff, in the unseen test set (n = 86,306).*

**

**Supplementary Table 5:** **Performance of the classifier of CH-HRG amongst Europeans.** Confusion matrix showing performance amongst those UK Biobank participants with self-reported European ancestry in the unseen test set.

**

**Supplementary Table 6:** **Performance of the classifier of CH-HRG amongst non-Europeans.** Confusion matrix of the performance amongst those UK Biobank participants with self-reported non-European ancestry in the unseen test set.

| **CHRS risk category** | **n** | **Median MN risk by MN predict** |
| --- | --- | --- |
| High | 100 | 34.3% |
| Intermediate | 418 | 7.9% |
| Low | 277 | 3.1% |

**Supplementary Table 7:** **Risk score categories amongst cases of CH-HRG.** The table summarises the distribution of risk scores by CHRS (Clonal Hematopoiesis Risk Score) and the median MN-predict ten-year MN risk within each of these categories.

| **Driver gene** | **Number by highest VAF** | **Number by any mutation** |
| --- | --- | --- |
| **JAK2** | 167 | 170 |
| **CALR** | 104 | 105 |
| **SF3B1** | 211 | 224 |
| **SRSF2** | 260 | 307 |
| **U2AF1** | 53 | 53 |
| **Total** | 795 | 859 |

**Supplementary Table 8: Variation in CH-HRG cases when labels are assigned by largest clone.** Number of participants with driver-gene specific CH when calling is based on the largest clone or the presence of any such driver mutation, where an individual harbours multiple mutations in different driver genes. The largest increase is in the number of individuals classified as harbouring SRSF2-CH.

***Supplementary Table 9:*** ***Hyperparameter values used in model optimisation.*** *Values were tuned using grid search, during model optimisation.*

***Supplementary Table 10****:* ***Proportions of "other" driver mutations in the UK Biobank final dataset.*** *Final dataset n=431,531.*

**References**

1. Sudlow, C. *et al.* UK biobank: an open access resource for identifying the causes of a wide range of complex diseases of middle and old age. *PLoS Med* **12**, e1001779 (2015).

2. Gu, M. *et al.* Multiparameter prediction of myeloid neoplasia risk. *Nat Genet* **55**, 1523–1530 (2023).

3. Karczewski, K. J. *et al.* The mutational constraint spectrum quantified from variation in 141,456 humans. *Nature* **581**, 434–443 (2020).

4. Vlasschaert, C. *et al.* A practical approach to curate clonal hematopoiesis of indeterminate potential in human genetic data sets. *Blood* **141**, 2214–2223 (2023).

5. Miller, C. A. *et al.* Failure to Detect Mutations in U2AF1 due to Changes in the GRCh38 Reference Sequence. *The Journal of Molecular Diagnostics* **24**, 219–223 (2022).

6. Danecek, P. *et al.* Twelve years of SAMtools and BCFtools. *Gigascience* **10**, giab008 (2021).

7. Liaw, A. & Wiener, M. Classification and Regression by RandomForest. *Forest* **23**, (2001).

8. Kuhn, M. Building Predictive Models in R Using the caret Package. *Journal of Statistical Software* **28**, 1–26 (2008).

9. Khoury, J. D. *et al.* The 5th edition of the World Health Organization Classification of Haematolymphoid Tumours: Myeloid and Histiocytic/Dendritic Neoplasms. *Leukemia* **36**, 1703–1719 (2022).

10. Kar, S. P. *et al.* Genome-wide analyses of 200,453 individuals yield new insights into the causes and consequences of clonal hematopoiesis. *Nat Genet* **54**, 1155–1166 (2022).
